# The Perception of the Diaphragm with Ultrasound: Always There Yet Overlooked?

**DOI:** 10.3390/life15020239

**Published:** 2025-02-05

**Authors:** Kathleen Möller, Max Saborio, Heike Gottschall, Michael Blaivas, Adrian C. Borges, Susanne Morf, Burkhard Möller, Christoph F. Dietrich

**Affiliations:** 1 Medical Department I/Gastroenterology, SANA Hospital Lichtenberg, 10365 Berlin, Germanyheike.gottschall@sana.de (H.G.); 2 Department General Internal Medicine (DAIM), Hospitals Hirslanden Bern Beau Site, Salem and Permanence, 3013 Bern, Switzerland; max.saborio@hirslanden.ch; 3 Department of Internal Medicine, School of Medicine, University of South Carolina, Columbia, SC 29209, USA; mike@blaivas.org; 4 Medical Department II/Cardiology, SANA Hospital Lichtenberg, 10365 Berlin, Germany; adrianconstantin.borges@sana.de; 5 Center da Sandà Val Müstair, 7536 Sta. Maria, Switzerland; susanne.morf@icloud.com; 6 Department of Rheumatology and Immunology, Bern University Hospital, Inselspital, University of Bern, 3010 Bern, Switzerland; burkhard.moeller@insel.ch

**Keywords:** diaphragm dysfunction, ultrasonographic diagnosis, diaphragm thickness, diaphragm thickening fraction, amplitude

## Abstract

Diaphragm ultrasound makes it possible to diagnose diaphragmatic atrophy and dysfunction. Important indications include unclear dyspnea; diaphragmatic elevation; assessment of diaphragm dysfunction in pulmonary, neuromuscular and neurovascular diseases; and in critically ill patients before noninvasive and mechanical ventilation and follow-up of diaphragm thickness and function during mechanical ventilation with potential prediction of prolonged weaning. In patients with respiratory insufficiency and potential diaphragm dysfunction, it is possible to objectify the contribution of diaphragm dysfunction. In addition, assessment of diaphragmatic hernias, tumors and diaphragmatic dysfunction in COVID-19 and diaphragmatic ultrasound in sports medicine have been described. This narrative review includes the sonomorphology of the diaphragm, standardization of ultrasonographic investigation with transducer positions and ultrasound techniques, normal findings and diagnostic criteria for pathological findings. The correct sonographic measurement, calculation and evaluation can ultimately influence further therapeutic procedures for the patient suffering from diaphragm dysfunction in various diseases.

## 1. Introduction

The thoracic diaphragm is a thin but important muscle of respiration that is frequently visible in whole or in part during ultrasound examinations of the abdomen and thorax but is perhaps overlooked or at least given too little attention. 

In critically ill patients, neuromuscular or neurovascular diseases and chronic lung diseases, functional limitations of the diaphragm may occur, leading to, for example, respiratory insufficiency. Trauma can lead to diaphragm injuries. 

Cardiopulmonary diagnostics are typically performed in the presence of dyspnea. If the findings are not conclusive or if there is a corresponding medical history and comorbidity, diaphragm dysfunction should also be taken into consideration. However, indications are that diaphragm ultrasound is not widely used. 

We describe the dysfunction of the diaphragm in various clinical scenarios. Even in healthy subjects, diaphragm measurement parameters vary depending on transducer position, phase of breathing and body position. A standardized examination and knowledge of these variations are important prerequisites for collecting and evaluating precise parameters. 

There are a large number of studies dealing with diaphragm parameters and dysfunction. However, diaphragm ultrasound could be used more widely in everyday clinical practice. This article presents a complete methodology of diaphragm ultrasound, normal parameters and their variation and describes the diagnosis of diaphragm dysfunction. Additionally, several important controversies are discussed.

This manuscript provides a comprehensive overview of ultrasound of the diaphragm and its applications. It is important to pay attention to this narrow structure, to know how the diaphragm is assessed during an ultrasound examination and to know which findings are normal and which are criteria for pathological deviations.

## 2. Diaphragm Anatomy

The diaphragm is a dome-shaped muscle that separates the abdominal cavity from the thoracic cavity. The central tendon of the diaphragm, a tendon around which the muscle fibers are arranged, is located centrally. Ventrally, the sternal part arises on the back of the xiphoid process. Dorsally, the lumbar part originates from the spinal column, and laterally, the costal part is attached to the inner sides of the cartilage of the lower six ribs. The diaphragm is the most important respiratory muscle and performs an estimated 75% of the inspiratory muscle work. When the diaphragm contracts during inspiration, the diaphragmatic dome flattens and the thoracic cavity enlarges. This creates negative pressure, which causes the lungs to expand. At the same time, the pressure in the abdominal cavity increases. The diaphragm is covered by the peritoneum on the abdominal side and by the parietal pleura on the thoracic side.

## 3. Diaphragm on Ultrasound

### 3.1. Morphological Features of the Diaphragm and Echogenicity on Ultrasound

**Figure 1:**
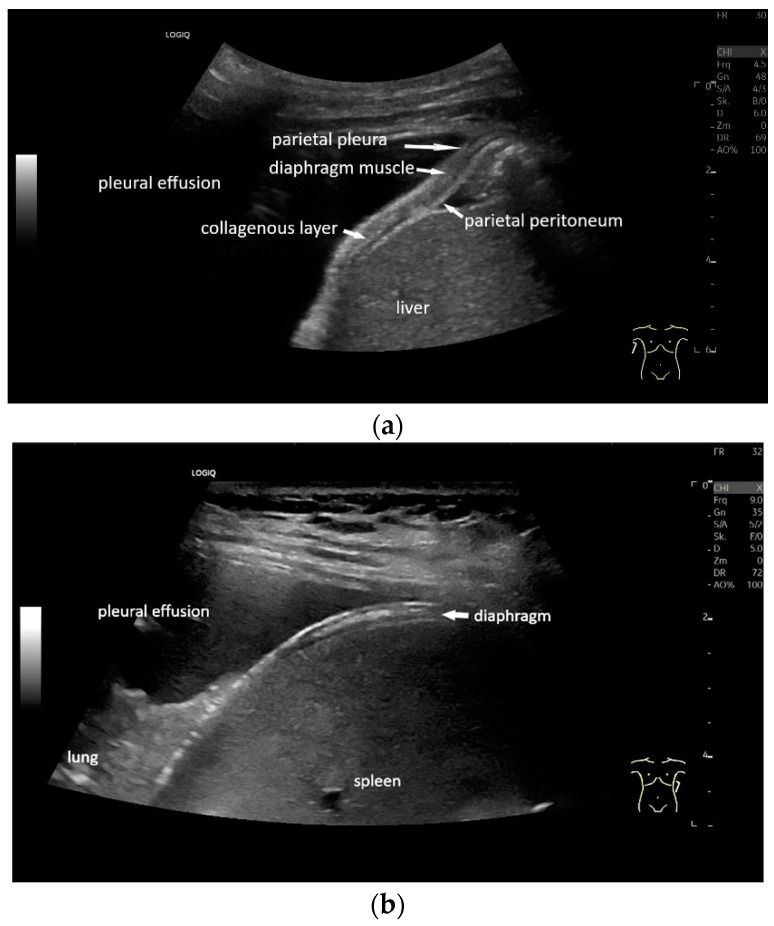
Diaphragm on the right (**a**) and left (**b**). Hypoechoic muscle, covered to the pleural cavity by the hyperechoic parietal pleura and to the abdomen by the hyperechoic peritoneum. In the center of the muscle there is another echogenic layer, which corresponds to narrow collagenous fibers. The diaphragm is particularly visible here, as it is not overlaid by lung artifacts in a pleural effusion.

With high-resolution linear transducers, the diaphragm presents itself as a hypoechoic band. It is covered on both sides by a very thin hyperechoic layer. This corresponds to the peritoneum and the parietal pleura, respectively. Depending on the resolution, a further delicate hyperechoic layer is usually visible centrally in the muscle, which is most often considered to be a fibrous layer in the center of the diaphragm (Figure 1).

#### Echogenicity—Dependence on Age, Gender, BMI, Transducer Type or Ultrasound Device

**Figure 2:**
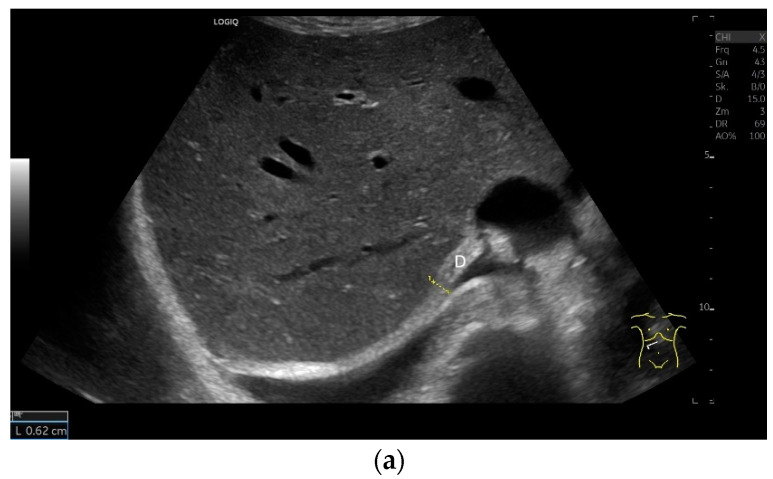

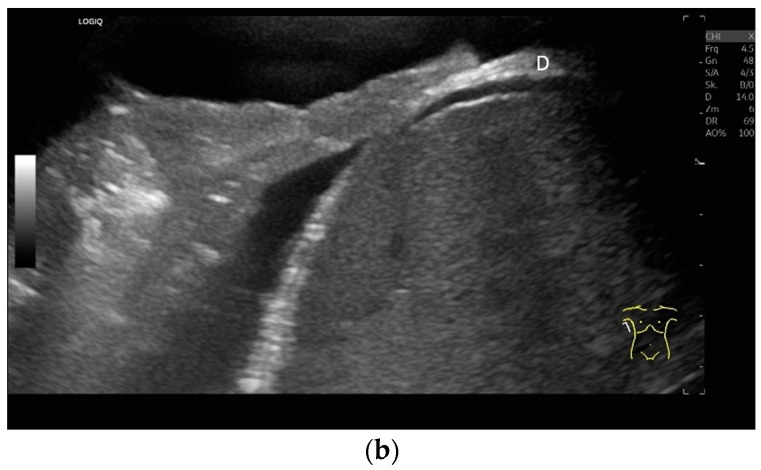
With abdominal sector transducers (**a**) and in elderly people (**b**), the diaphragm can appear hyperechoic. D—diaphragm.

The diaphragm is a hypoechoic muscular structure, the echogenicity of which increases with age. An increased body mass index (BMI) can also lead to an increase in diaphragm echogenicity. However, gender has no influence on echogenicity [1]. With abdominal sector transducers (2–5 MHz), the diaphragm can appear as a hyperechoic line. Overall, the echogenicity of the same patient may differ with different devices [1] (Figure 2).

### 3.2. Optimal Sonographic Techniques

#### Sonographic Adjustment and Transducer Positions

Examination is possible in a supine body position, with the upper body slightly elevated or in a seated or standing position. In our own experience, examination in a supine position is the most pragmatic. In particular, this applies to patients in intensive care or those on ventilators. If the supine position does not allow sufficient assessment, other positions can be tried depending on the individual patient’s situation. Supine positioning may not be possible for patients with respiratory insufficiency. 

The diaphragm can be assessed in two main transducer positions: lateral in the zone of apposition and subcostal. The zone of apposition is the section where the diaphragm is attached to the inside of the ribs. Measurements are taken in these both positions. In addition, the diaphragm can also be visualized subxiphoidally in the epigastrium in cross section. Depending on the depth of penetration, the examination is performed with an abdominal sector or cardiac transducer (2–5 MHz) or with high-resolution linear transducers. The choice of transducer depends on the depth of the penetration. Some authors choose transducers up to 15 MHz. We prefer a linear transducer up to 9 MHz. The assessment is conducted in B-mode ultrasound (US) and in M-mode US. 

**Figure 3:**
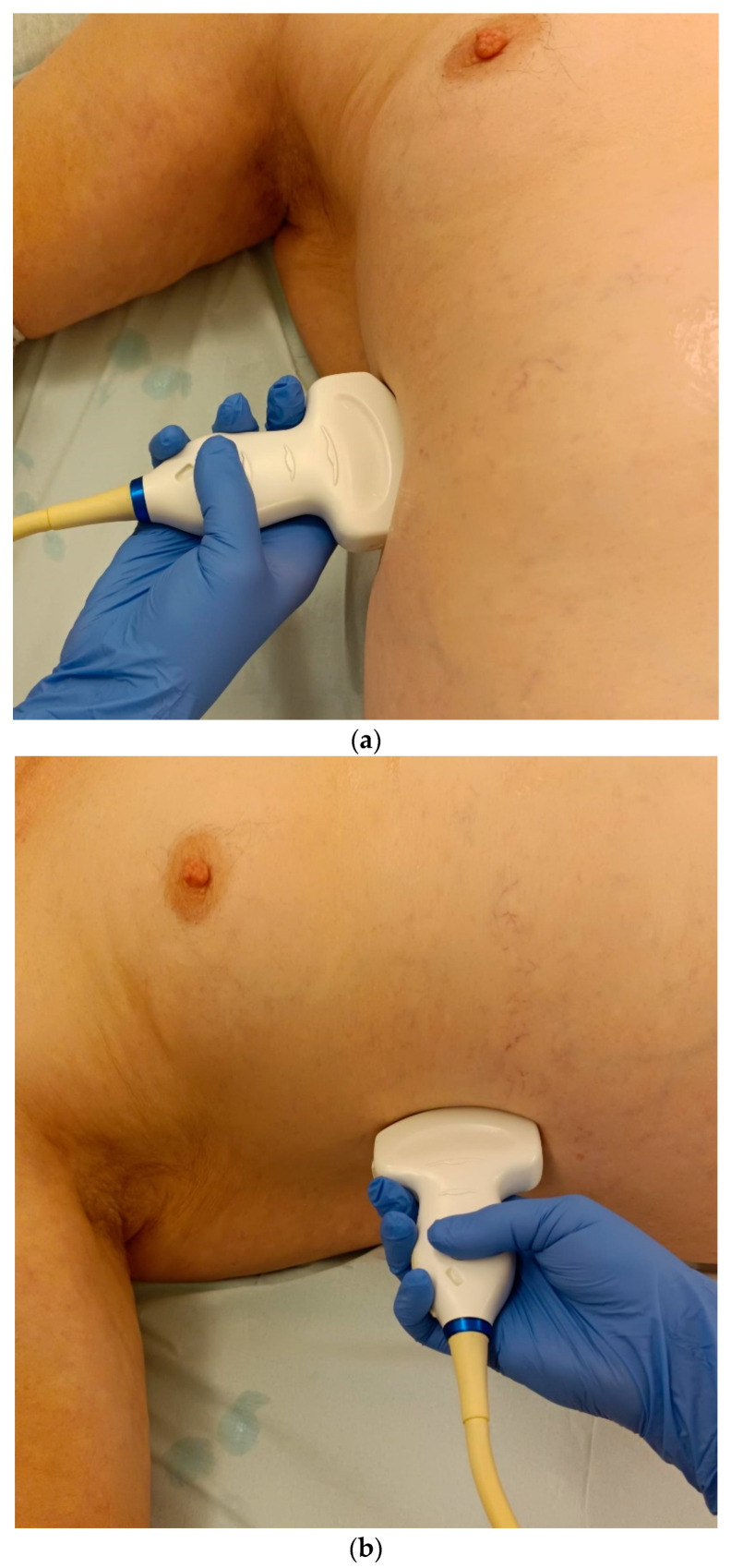

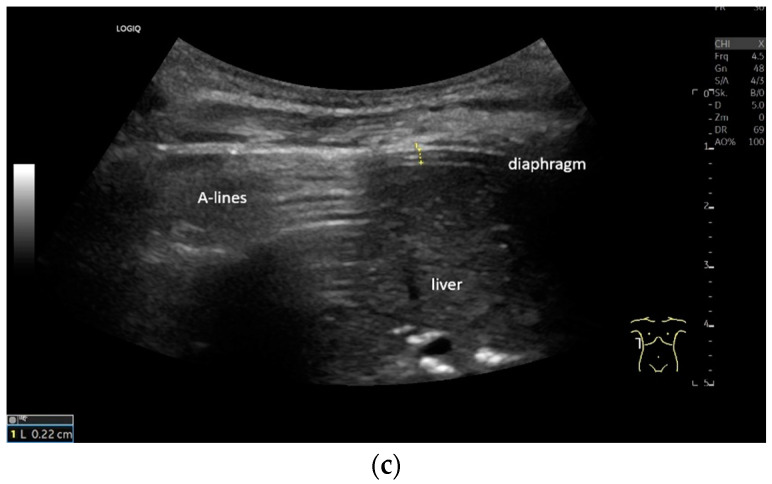
Transducer positions for visualization of the apposition zone on the right. The transducer is positioned longitudinally and laterally in the area of the mid-axillary line or slightly ventrally between the anterior and mid-axillary line, approximately in the 8th or 10th intercostal space (**a**,**b**). The diaphragm is located caudal to the pulmonary glide (typical A-lines/reverberation artifacts) (**c**). It is located on the inside of the intercostal spaces.

**Figure 4:**
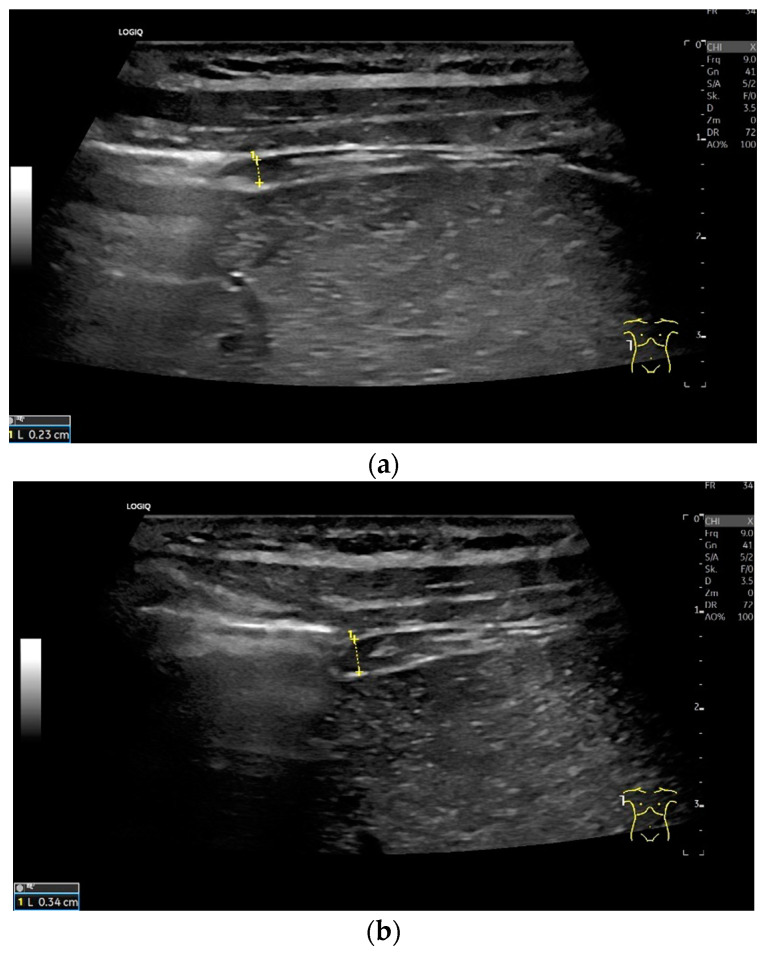

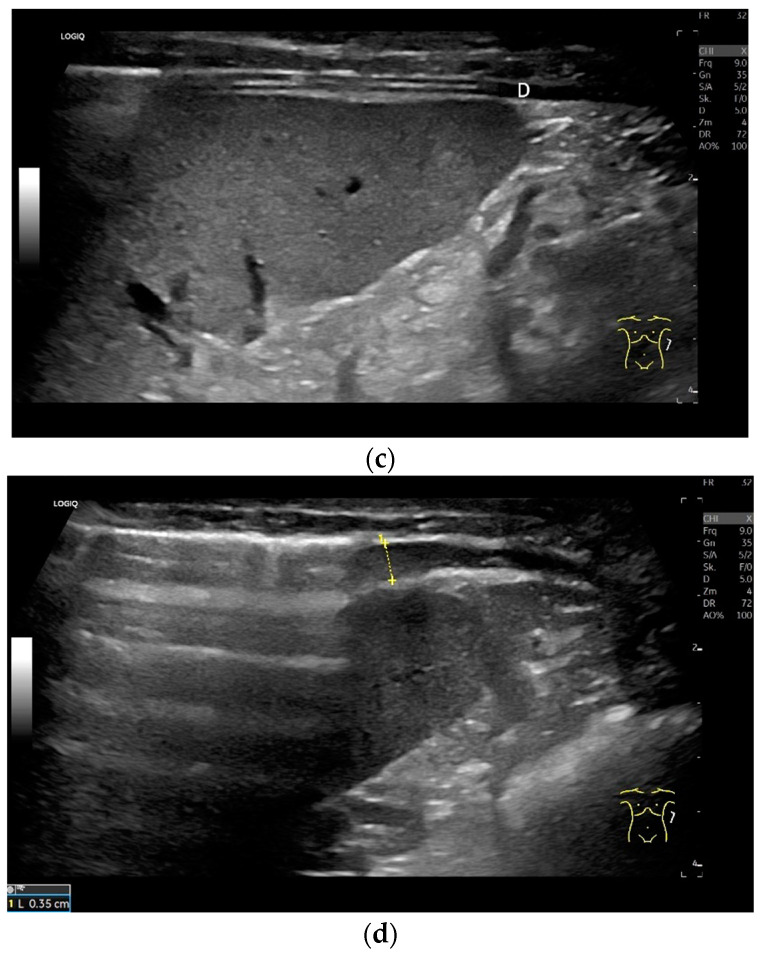
Measurement of the diaphragm thickness on the right and left in the apposition zone (9 MHz linear transducer). The markers are positioned within the peritoneum and the pleura. They are located on the outer contour of the hypoechoic muscle (**a**,**b**). The delicate hypoechoic collagenous central fiber is also visible. The diaphragm is thinner in the end-expiratory phase (**a**) than in the contracted end-inspiratory phase (**b**). The same measurements are performed on the left in the apposition zone. The diaphragm between the intercostal spaces and the spleen can be seen below the pulmonary glide, thinner at end-expiration phase (**c**) and during end-inspiratory contraction (**d**). D—diaphragm.

***Lateral transducer position in the zone of apposition***: The transducer is positioned longitudinally and laterally in the area of the mid-axillary line or slightly ventral to it between the anterior and mid-axillary lines, approximately in the 8th or 10th intercostal space. The diaphragmatic reflex is located on the inner side of the ribs below the pulmonary glide with pulmonary reverberations. This localization of origin of the diaphragm from the inner side of the rib cartilage is referred to as the zone of apposition (Figure 3). As this localization is only a few centimeters below the skin surface, high-resolution linear transducer use is highly recommended. In this position, the diameter of the diaphragm is measured during inspiration and expiration [2,3,4,5] (Figure 4). The thickness of the diaphragm varies, with caudal parts being thicker than cranial parts. The measurement of diaphragm thickness is highly variable depending on the intercostal space chosen, with thickness varying by up to 6 mm between the intercostal spaces [6]. It is therefore important to select the same position for comparable measurements and, if necessary, to mark the location for the transducer position [7]. Obesity limits assessment of the diaphragm [2].

**Figure 5:**
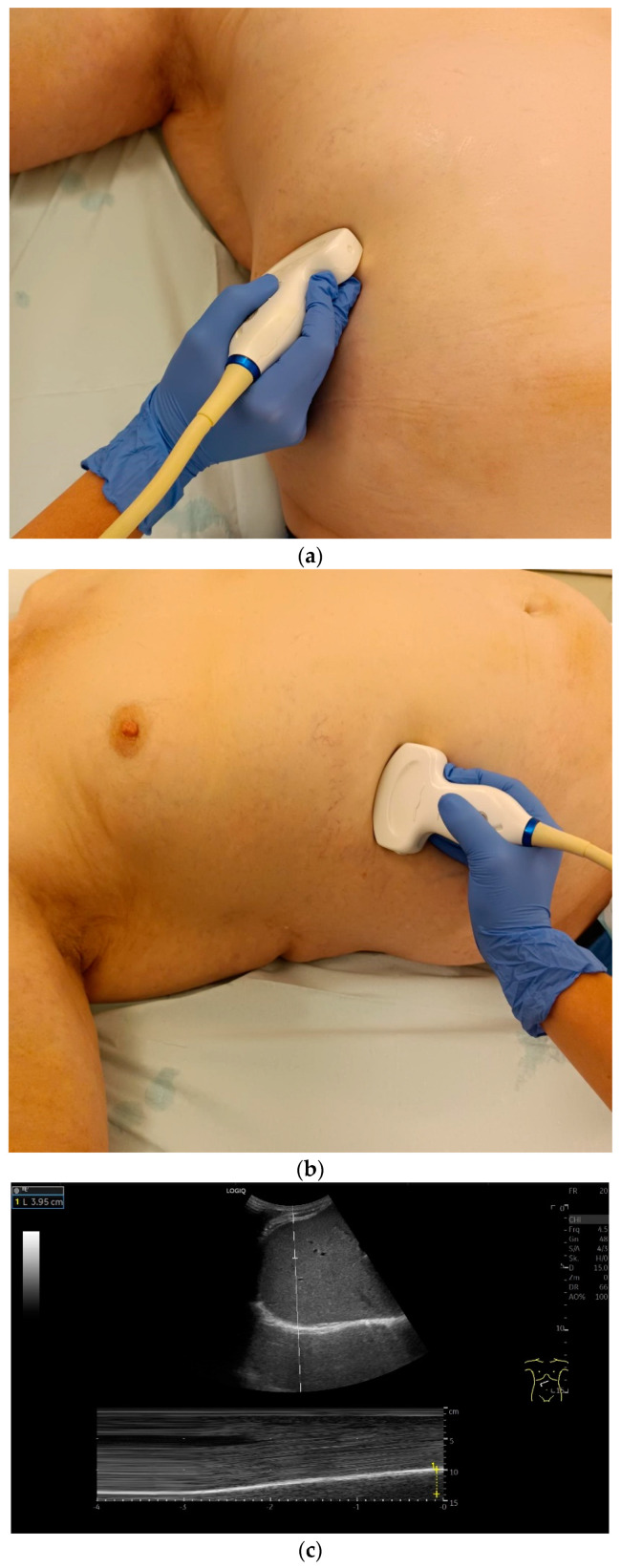

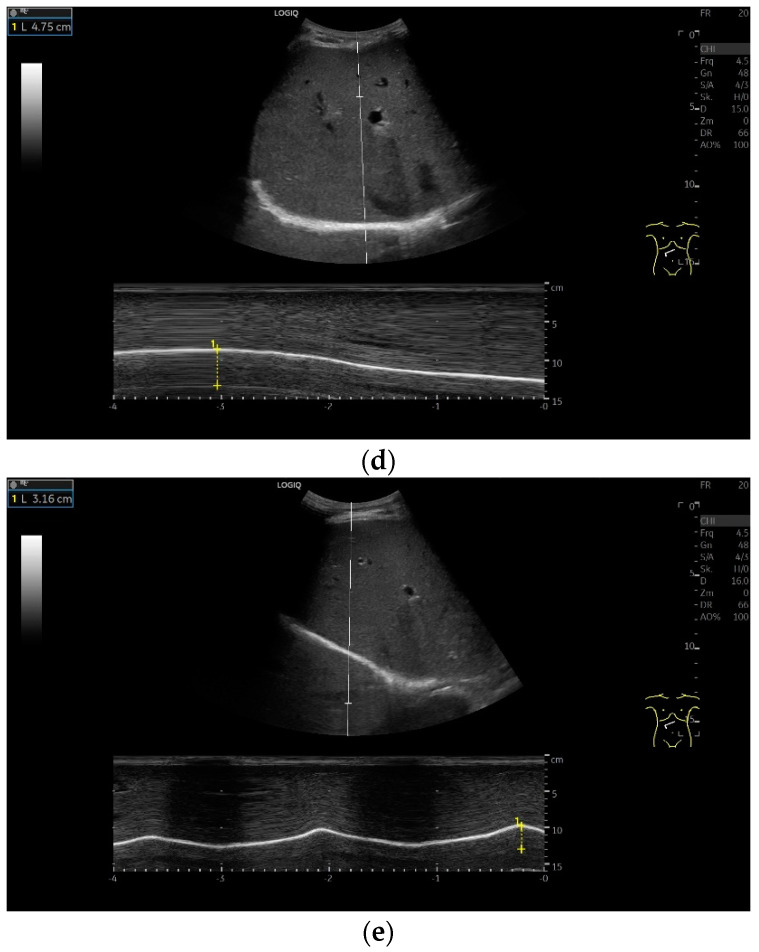
Subcostal transducer positions. The transducer is placed between the medioclavicular line and the anterior axillary line (**a**,**b**). The diaphragm excursion can then be visualized and measured in M-mode during breath holding and deep inspiration (**c**,**d**) and voluntary “sniffing” or coughing (**e**).

***Subcostal transducer position:*** An abdominal sector or cardiac transducer (2–5 MHz) is used in the subcostal window. This position is used to assess diaphragmatic excursion. The transducer is placed between the linea medioclavicularis and linea axillaris anterior. The diaphragmatic excursion can then be visualized and measured in M-mode (Figure 5). The gallbladder and inferior vena cava are important landmarks. It is important to guide the ultrasound probe as perpendicularly as possible. The M-mode axis should meet the diaphragm at an angle of 90%. In this position, diaphragm thickness in inspiration and expiration can also be measured using M-mode, and diaphragm shortening can be calculated. However, in normal adults, the diaphragm can usually only be visualized in this position using abdominal sector transducers and is less accurately delineated than with a linear transducer from the lateral side in the anterior axillary line.

**Figure 6:**
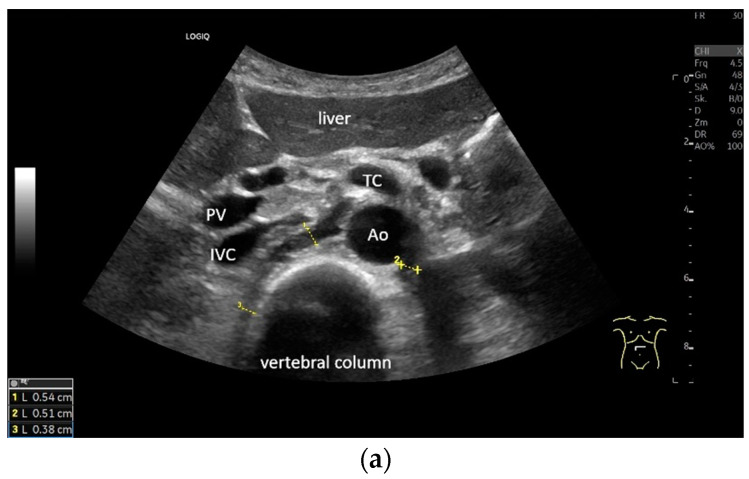

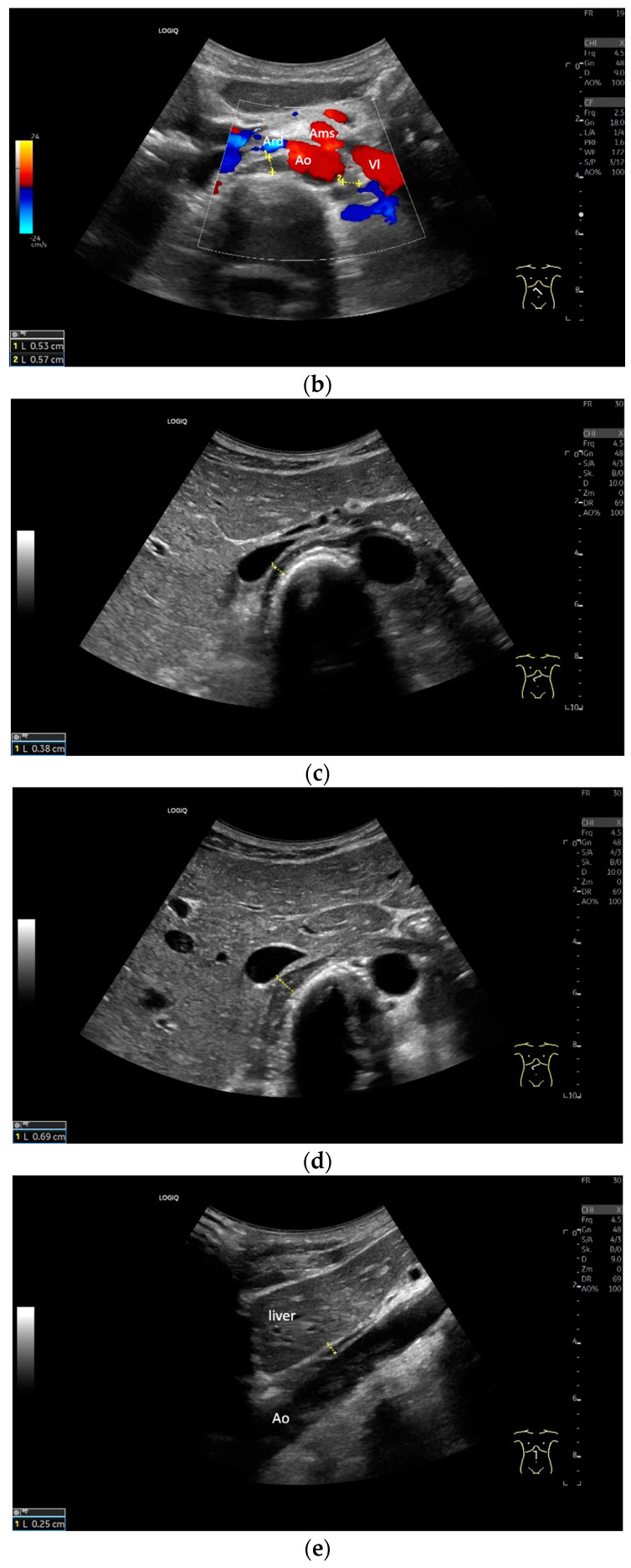

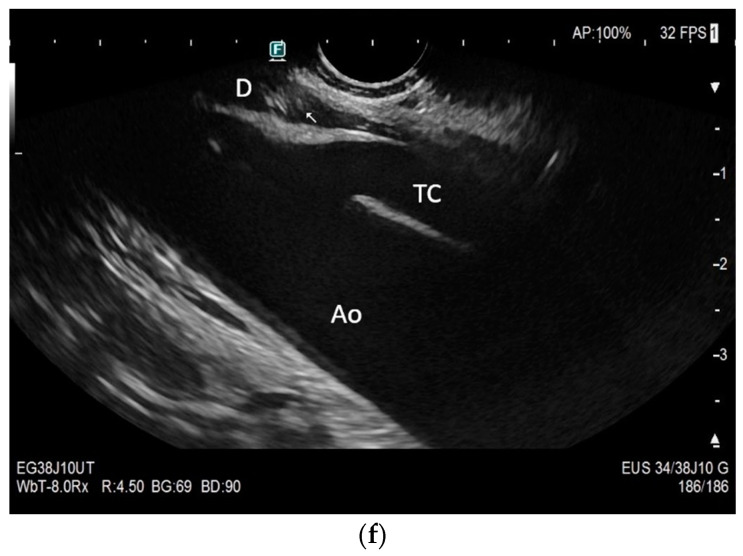
Subxiphoid transducer positions. The right diaphragmatic fat limb runs between the inferior vena cava and the aorta to the right of the spine. The left diaphragmatic limb is shown to the left of and dorsal to the aorta. Diaphragm between the markings (**a**). The diaphragm in the color Doppler imaging (CDI) between the vessels. The incisions should not be confused with lymphomas (**b**). The diaphragm on the right between the inferior vena cava and the spine (between the markings) and in front of the abdominal aorta (**c**). The thickened diaphragm during inspiration (between the markings) in the same position. Under optimal examination conditions, the contraction can also be visible in this position (**d**). The diaphragm between the aorta and liver is visible in longitudinal section (**e**). Endosonography shows the diaphragmatic limb ventral to the aorta (arrow) (**f**). Ams—superior mesenteric artery, Ao—aorta, Ard—right renal artery, D—diaphragm; IVC—vena cava inferior, PV—portal vein, TC—celiac trunk, Vl—splenic vein.

***Subxiphoid*:** The diaphragm can be positioned directly subxiphoidally with a slightly sagittal transducer position on both sides next to the attachment to the sternum. The diaphragm limb can be demarcated to the right in the epigastrium in cross section between the aorta and the inferior vena cava. However, this is primarily of differential diagnostic importance, e.g., in relation to lymphomas, not for the actual assessment of the diaphragm (Figure 6).

**Figure 7:**
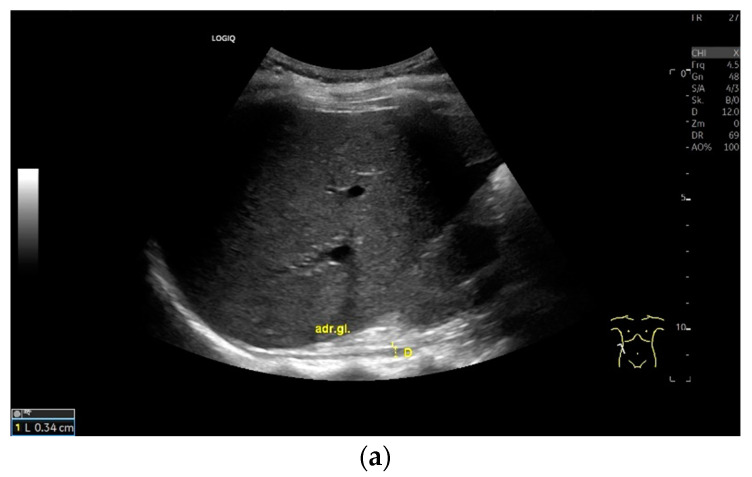

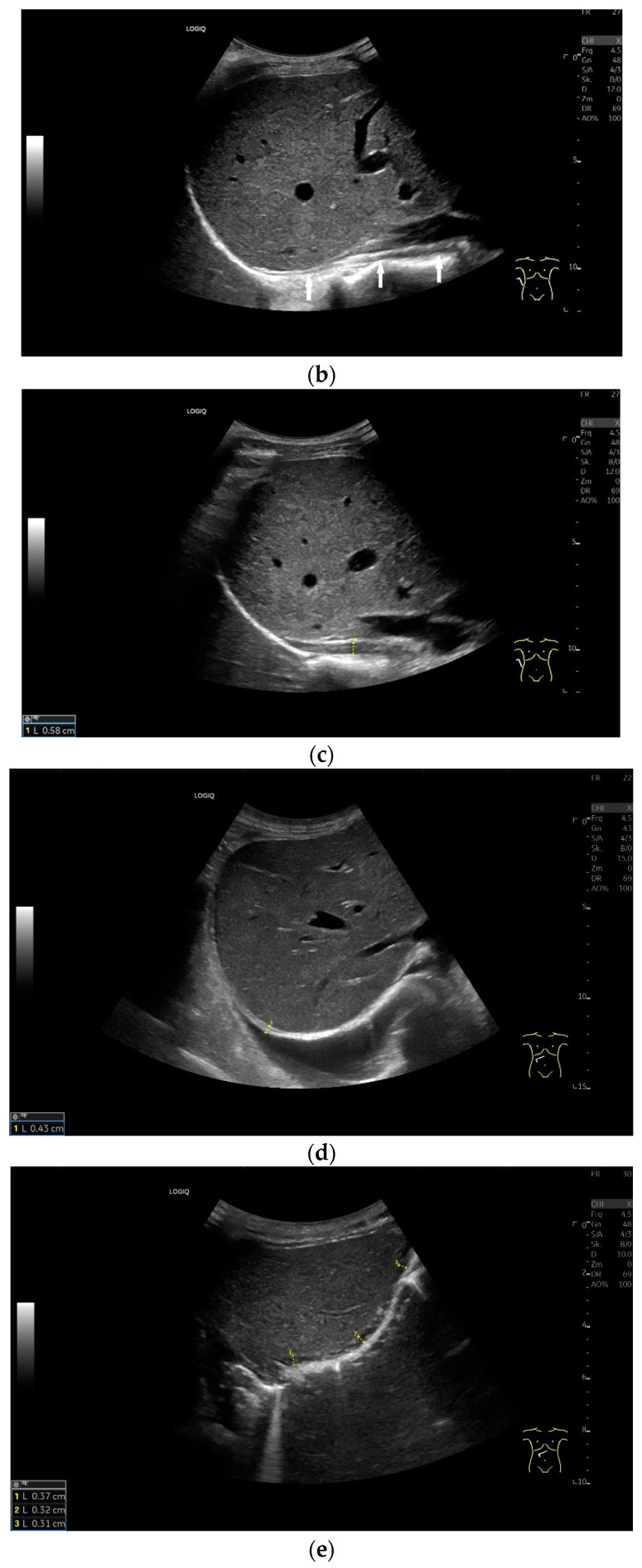

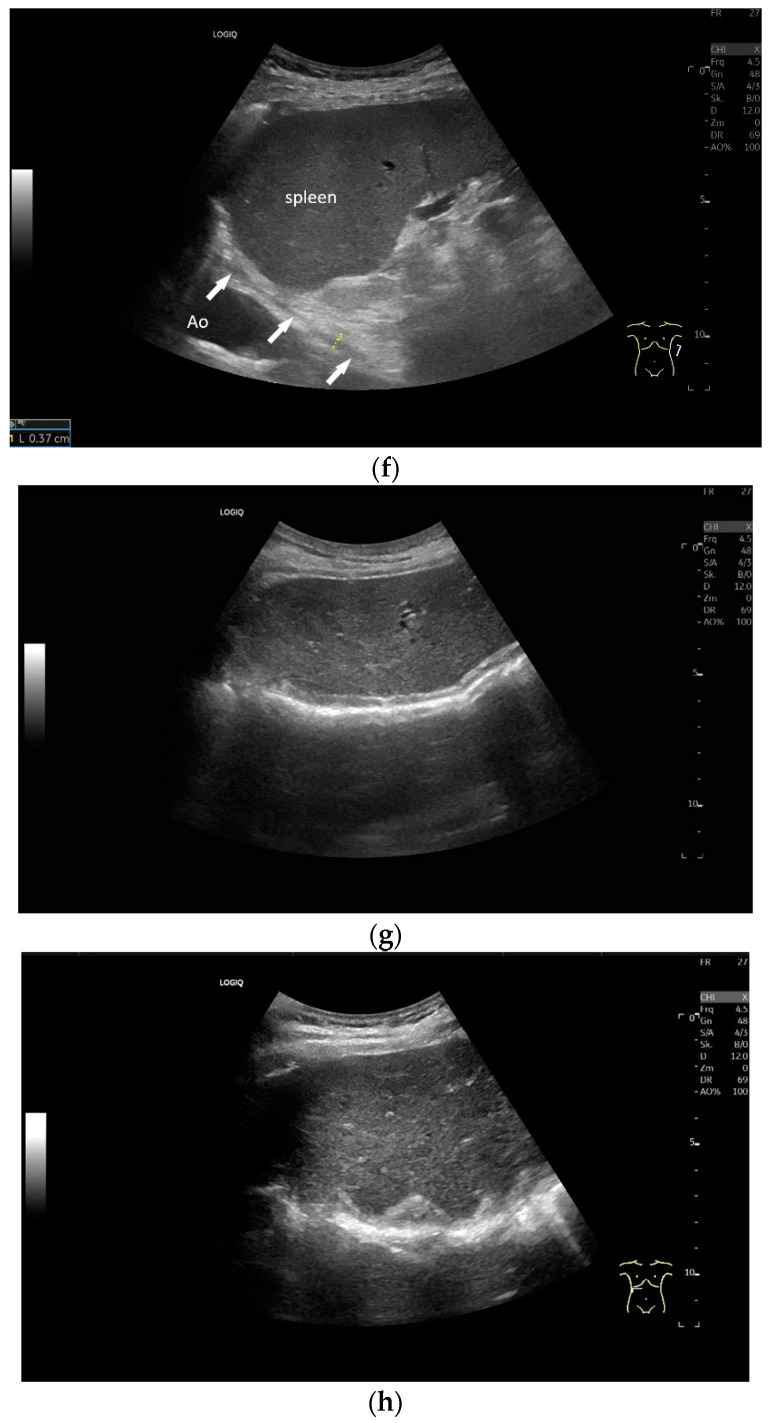
When imaging the right adrenal gland, the diaphragm is in one orientation. The diaphragmatic limb passes dorsal to the right adrenal gland and ventral to the vertebral column (**a**). The diaphragm in expiration ventral to the vertebral column with transducer position in the right flank longitudinal (arrows) (**b**) and thickened in inspiration (**c**). Diaphragm with typical triple layering in the subcostal section in the medioclavicular line, well delineated in pleural effusion (**d**). The transducer is directed medially in the MCL (**e**). In the case of pleural effusion and lack of total lung reflection, the diaphragm can also be seen in a longer extension on the left side (**f**). Mirror reflection of the diaphragm. Hyperechoic peritoneum, hypoechoic diaphragm muscle, hyperechoic total reflection of the lung. The hyperechoic parietal pleura cannot be differentiated from the total reflection of the lung. This also applies to the mirrored side. In the area of the mirror artifact are the hypoechoic diaphragmatic muscle and the hyperechoic parietal peritoneum. The diaphragm is not thickened, and the total reflection of the lung should not be confused with the collagenous layer (**g**). Diaphragmatic incisions (**h**).

Regardless of the recommended positions for standardized measurements, the diaphragm can be viewed in other areas. This works quite well on the flank and subcostally in the midclavicular line (Figure 7).

Some recommendations for the performance of diaphragm ultrasound in critically ill patients are summarized in consensus guidelines and correspond to the procedures described here [4]. Measurements on the right side are acceptable to assess overall diaphragmatic function. However, if unilateral diaphragmatic pathology is suspected following thoracic surgery, phrenic nerve injury or spinal cord injury, measurements must be taken on both sides [4].

### 3.3. Clinical Parameters and Normal Values

#### 3.3.1. Important Parameters to Measure

To assess the diaphragm and its function, the diaphragm’s thickness, contraction and excursion are determined. Diaphragm thickness is mainly measured in the zone of apposition and excursion in the subcostal position. The diaphragm thickness is measured at end-expiratory (T_endexpir_) and end-inspiratory (T_endinspir_) points. Expiratory and inspiratory measurements should be taken with exactly the same transducer position. The measurement is taken at the outer edges of the hypoechoic muscle and at an angle of 90° to the diaphragm’s surface (Figure 4).

The diaphragm thickening ratio (TR_di_) is calculated from these two parameters: thickness at end-inspiration divided by the thickness at end-expiration [8]. The reference value corresponds at 2.1 to 2.2 for quiet respiration [3,8]. In practice, this means that the end-inspiratory diaphragm is slightly more than twice as thick as the end-expiratory diaphragm. The diaphragm thickening fraction (TF_di_) corresponds to the percentage increase in diaphragm thickness during inspiration: (end-inspiratory diaphragm thickness−end-expiratory diaphragm thickness)/end-expiratory diaphragm thickness × 100. The reference value is >36–37% in quiet breathing [9,10]. The calculations can be based on both B-mode and M-mode data of T_endexpir_ and T_endinspir_.

Diaphragmatic excursion and amplitude reflect the range of mobility and contraction. They can be assessed using different breathing maneuvers: during quiet breathing, with deep inspiration and during sniffing. A sniff maneuver is a short, forced inspiration. The movement of the diaphragm is ideally mapped in M-mode. The amplitude of diaphragmatic movement can then be measured on the M-mode curve, as can the speed of the diaphragm excursion. Standard values in numerous studies for diaphragm thickness, contraction and movement amplitude depending on body position, gender and diaphragm side are listed in Table 1 and Table 2. In addition to mean values and standard deviations, various studies also report the lower limit of normality of diaphragm thickness and contraction as well as the derived parameters. These are limit values that indicate how high a parameter must be in order not to be considered pathological. In this way, patients with dysfunction can be identified. There is also information on the upper limit of normality. However, the meaning of such a value is not entirely clear. 

In the consensus guidelines for diaphragm ultrasound in critically ill patients, it is estimated that 40 diaphragm ultrasound examinations should be performed to enable independent examination in daily practice. Ideally, these should be bilateral, 20 of which should be performed under supervision. The consensus authors felt that diaphragmatic excursion is easy to examine, while thickness measurement takes longer to learn [4].

#### 3.3.2. Normal Values

Normal values in healthy individuals are shown in Table 1.

**Table 1:** Normal values in healthy individuals.

Study	Parameter	All	Male	Female	Additional Information
Gottesman 1997 [9]; n = 15 healthy; **standing** position; 7.5–10 MHz	T_di_ at end-expiration (FRC)	2.8 ± 0.4 mm			No significant difference between right and left diaphragm.
	Diaphragm thickening ratio (TR_di_)	37 ± 9% (range 21–57%)			
Carillo-Esper 2016 [11]; n = 109 healthy; **supine** position; 5–10 MHz	Tdi at end-expiration (FRC)	1.6 ± 0.4 mm (95% CI 1.5–1.7 mm)	1.9 ± 0.4 mm (95% CI 1.7–2.0 mm) LLN 1.7 mm	1.4 ± 0.3 mm (95% CI 1.3–1.5 mm) LLN 1.4 mm	No relation between BMI, thorax circumference and diaphragmatic thickness. Significant difference in the diaphragmatic thickness between genders.
Cardenas (2018) [12]; n = 65 healthy; **semi-recumbent position** with the bed slope at 45°; 2–5 MHz; 6–13 MHz	T_di_ at end-expiration (FRC)		1.87 ± 0.31 mm LLN 1.25 mm	1.79 ± 0.28 LLN 1.23 mm	LLNs were calculated.
	T_di_ at end-inspiration (TLC)		5.59 ± 0.88 mm LLN 3.83 mm	4.81 ± 0.95 mm LLN 2.91 mm	
	Diaphragm thickening fraction TF_di_ (%)		204.23 ± 61.96% LLN 80.31%	169.67 ± 43.67% LLN 82.33%	
Scarlata 2019 [13]; n = 66 healthy; **recumbent position**; linear probe	T_di_ at end-inspiration (TLC)	2.6 ± 0.5 mm			
	T_di_ at end-expiration (FRC)	1.8 ± 0.4 mm			
Spiesshoefer 2020 [3]; n = 70 healthy; **supine** position; 3.5 MHz/10 MHz	T_di_ at end-expiration (FRC) Lower limit of normal (LLN) of the 95% CI around the mean	1.9 ± 0.6 mm	2.2 ± 0.8 mm LLN 1.7 mm	1.8 ± 0.5 mm LLN 1.5 mm	The lower limit of normal of the 95% CI around the mean was calculated. Values in males were higher than in females. There was no age-related correlation.
	T_di_ at end-inspiration (TLC)	5.3 ± 1.8 mm	6.3 ± 1.7 mm LLN 4.6 mm	4.7 ± 1.7 mm LLN 3.5 mm	
	Diaphragm thickening ratio (TR_di_)	2.86 ± 0.88 mm	3.03 ± 0.95 LLN 2.2 mm	2.77 ± 0.83 LLN 2.2 mm	
Boussuges 2021 [8]; n = 200 healthy; **seated** position; 9 MHz	T_di_ at end-expiration (FRC) right hemidiaphragm		2.1 ± 0.4 mm [1.3, 3.0]	1.9 ± 0.4 mm [1.1, 2.7]	Lower limit and upper limit of normality in [brackets].
	T_di_ at end-expiration (FRC) left		2.0 ± 0.4 mm [1.3, 2.7]	1.7 ± 0.3 mm [1.1, 2.4]	
	T_di_ at end-inspiration (TLC) QB right		2.8 ± 0.6 mm [1.7, 3.9]	2.5 ± 0.6 mm [1.3, 3.7]	
	T_di_ at end-inspiration (TLC) QD left		2.6 ± 0.5 mm [1.7, 3.5]	2.3 ± 0.5 mm [1.3, 3.3]	
	T_di_ at end-inspiration (TLC) DB right		4.3 ± 0.8 mm [2.8, 5.9]	3.9 ± 0.8 mm [2.4, 5.4]	
	T_di_ at end-inspiration (TLC) DB left		4.2 ± 0.8 mm [2.6, 5.8]	3.8 ± 0.8 mm [2.3, 5.3]	
	TR_di_ right		2.1 ± 0.3 [1.4, 2.7]	2.2 ± 0.4 [1.4, 2.9]	
	TR_di_ left		2.1 ± 0.4 [1.4, 2.8]	2.2 ± 0.4 [1.5, 2.9]	
	TF_di_ right		106 ± 34% [40, 173]	116 ± 40% [39, 193]	
	TF_di_ left		112 ± 37% [39, 184]	121 ± 37% [48, 193]	
Yamada 2024 [14]; 109 healthy; **seated** position; 7.0 MHz	T_di_ at end-expiration (FRC); right	1.7 ± 0.4 mm [0.9, 2.5]	1.8 ± 0.3 mm [1.2, 2.4]	1.7 ± 0.4 mm [0.6, 2.6]	Lower limit and upper limit of normality in [brackets].
	T_di_ at end-expiration (FRC); left	1.6 ± 0.4 mm [0.8, 2.4]	1.7 ± 0.4 mm [0.9, 2.5]	1.5 ± 0.4 mm [0.7, 2.3]	
	TF_di_/QB; right	50.0 ± 25.9%	46.4 ± 20.8%	53.8 ± 30.0%	
	TF_di_/QB; left	51.8 ± 26.9%	49.5 ± 23.6%	54.2 ± 30.0%	
	TF_di_ DB; right	110.7 ± 44.3% [24.3, 197.1]	113.8 ± 46.0% [24.1, 203.5]	107.4 ± 42.6% [24.3, 190.5]	
	TF_di_ DB; left	107.2 ± 43.8% [21.8, 192.6]	108.6 ± 42.7% [25.3, 191.9]	105.6 ± 45.4% [17.1, 194.1]	
Zhang 2024 [15]; 212 healthy; **supine** position; 3–11 MHz linear array probe; 1–6 MHz convex array probe	T_di_ at end-expiration (FRC)	1.4± 0.5	1.6 ± 0.5	1.2 ± 0.4	
	T_di_ at end-inspiration (TLC)	2.9 ± 1.0	3.2 ± 1.0	2.6 ± 0.9	

Legend: Functional residual capacity (FRC) is the volume remaining in the lungs after a normal, passive exhalation. Total lung capacity (TLC) is the volume of air in the lungs at the end of maximal inspiration. DB, deep breathing; QB, quiet breathing; T_di_, diaphragm thickness; TR_di_, thickening ratio; TF_di_, thickening fraction; LLN, lower limit of normality; ULN, upper limit of normality.

**Table 2:** Diaphragm mobility in healthy volunteers.

Study	Parameter	All	Male	Female	Additional Information
Cardenas 2018 [12]; n = 65 healthy; **semi-recumbent** position with the bed slope at 45°; 2–5 MHz; 6–13 MHz	Diaphragmatic amplitude/QB		1.52 ± 0.43 LLN 0.66 cm	1.41 ± 0.32 LLN 0.77 cm	QB: mobility, thickness (ThFRC) without differences between genders. DB: mobility, thickness (ThTLC); TF_di_ values were lower in females.
	Diaphragmatic amplitude/DB		7.79 ± 0.82 LLN 6.15 cm	6.41 ± 1.02 LLN 4.37 cm	
	Diaphragm amplitude during tidal breathing	1.56 ± 0.53 cm	1.71 ± 0.61 LLN 1.2 cm	1.47 ± 0.46 LLN 1.2 cm	
	Amplitude during voluntary sniff	2.52 ± 1.00 cm	2.96 ± 1.23 LLN 2.0 cm	2.28 ± 0.76 LLN 1.5 cm	
	Amplitude during max. inspiration	8.02 ± 1.91 cm	9.07 ± 1.94 LLN 7.9 cm	7.48 ± 1.58 LLN 6.4 cm	
	Velocity during tidal breathing	1.12 ± 0.44 cm/s	1.23 ± 0.56 LLN 0.8 cm/s	1.06 ± 0.35 LLN 0.8 cm/s	
	Velocity during voluntary sniff	6.82 ± 2.03 cm/s	8.16 ± 1.85 LLN 6.7 cm/s	6.08 ± 1.74 LLN 5.2 cm/s	
Scarlata 2018 [16]; n = 108 healthy; **recumbent**; 2.5 and 3.5 MHz	Diaphragm amplitude/QB	1.76 ± 0.54 cm	2.03 ± 0.57 cm	1.51 ± 0.37 cm	Positive correlation of diaphragm motion with height and weight in QB and DB. Negative Correlation with age in DB.
	Diaphragm amplitude/DB	6.20 ± 1.55 cm	6.93 ± 1.46 cm	5.54 ± 1.33 cm	
Boussuges 2021 [8]; n = 200 healthy; **seated** position; 9 MHz	Right excursion/QB		2.0 ± 0.5 cm	1.9 ± 0.5 cm	
	Right excursion/voluntary sniffing		2.7 ± 0.7 cm	2.3 ± 0.7 cm	
	Right excursion/DB		6.0 ± 0.9 cm	5.0 ± 0.9 cm	
	Left excursion/QB		2.2 ± 0.6 cm	1.9 ± 0.5 cm	
	Left excursion/voluntary sniffing		2.8 ± 0.8 cm	2.4 ± 0.6 cm	
	Left excursion /DB		6.2 ± 0.9 cm	5.0 ± 0.7 cm	
Yamada 2024 [14]; 109 healthy; **seated** position; 7.0 MHz	Diaphragm excursion/QB/right hemidiaphragm	1.7 ± 0.6 cm [0.5, 2.9]	1.8 ± 0.6 cm [0.6, 3.0]	1.6 ± 0.6 cm [0.4, 2.8]	Lower limit and upper limit of normality in [brackets].
	Diaphragm excursion/QB/left hemidiaphragm	1.9 ± 0.7 cm [0.5, 3.3]	2.0 ± 0.7 cm [0.6, 3.4]	1.7 ± 0.6 cm [0.5, 2.9]	
	Diaphragm excursion/DB/right hemidiaphragm	4.4 ± 1.4 cm [1.7, 7.1]	5.1 ± 1.3 cm [2.6, 7.6]	3.8 ±1.1 cm [1.7, 6.0]	
	Diaphragm excursion/DB/left hemidiaphragm	4.1 ± 1.1 cm [2.0, 6.3]	4.7 ± 1.1 cm [2.6, 6.9]	3.8 ± 1.0 cm [1.9, 5.8]	
Zhang 2024 [15]; 212 healthy; **supine** position; 3–11 MHz linear array probe; 1–6 MHz convex array probe	Diaphragm excursion QB (cm)	1.69 ± 0.37 cm	1.75 ± 0.37 cm	1.63 ± 0.35 cm	
	Diaphragm excursion DB (cm)	5.06 ± 1.40 cm	5.05 ± 1.36 cm	5.07 ± 1.45 cm	

Legend: DB, deep breathing; QB, quiet breathing.

##### 3.3.3. Limit Values

It is difficult to recommend reference values for everyday clinical practice because study data differ, and different reference values are given in several studies. Ultimately, the diaphragm’s thickness depends on gender, BMI and patient and transducer positioning. The lower limit of normality (LLN) represents important limit values for diaphragm thickness (T_di_), thickening ratio (TR_di_), thickening fraction (TF_di_) and amplitude, and the upper limit of normality (ULN) represents important limit values for the difference between the two diaphragm hemispheres (Table 3).

##### 3.3.4. Diaphragm Parameters as a Function of Gender, Age, BMI and Body Position

The diaphragm is slightly thicker in men than in women [3,8,11,12,15]. However, this difference is not always statistically significant [14]. Nevertheless, gender-specific normal values should be used for diaphragm thickness interpretation. Some studies have found a correlation between diaphragm thickness and body mass index (BMI) [1,6,8,14]. Diaphragm thickness end-expiratory and end-inspiratory were positively correlated with age [15]. Diaphragm excursion during quiet breathing (QB) was negatively correlated with age and BMI [15]. Yamada et al. [14] obtained lower diaphragm thicknesses in healthy Japanese subjects with lower BMI than in European studies. They concluded that BMI does have an impact on diaphragm thickness and is the reason why lean Japanese subjects in the study had a lower diaphragm thickness than healthy subjects in European studies. The thickening fraction during deep breathing (DB) was not affected [14]. Boussuges et al. found no significant difference for TF_di_ in QB and deep breathing for both sexes [8].

The diaphragm excursion/amplitude is greater in men than in women [8,12,13,14,16]. In the study by Scarlata 2019 et al. [13], body size and weight did not differ between genders, so in the opinion of the authors, other factors must also be discussed as causes for different diaphragm excursions in males and females. The authors also found no correlation between thickness, TR_di_, TF_di_ and anthropometric data such as age, weight, height and BMI. It is discussed that factors such as physical fitness and regular exercise must also have an impact.

The examination position can also influence the measured values. Diaphragm thickness and end-inspiratory thickening were more than 20% thicker in the seated and standing positions than in the supine position at maximal inspiration/TLC [18]. However, when comparing different studies in which diaphragm thickness in end-expiration was measured in either the supine [3,11] or seated [8,14] position, diaphragm thickness was greater in the supine position than in the seated position. 

In another study, the normal percentage thickening of the diaphragm was significantly greater in the upright position than when supine. In supine positions it was 60%, while it was 97% when seated and 174% when standing [19]. The ultrasound examination identified differences in the contractility of the diaphragm due to body position. The effects of gravity on diaphragmatic tension and the changes in intra-abdominal pressure due to body position could explain the differences detected. This is important information for patients undergoing rehabilitation after intensive medical treatment [19]. The amplitude of the diaphragmatic excursion also depends on the body position. In contrast to TF_di_, the amplitude is greater in the supine position than in the seated position [17].

##### 3.3.5. Right and Left Hemidiaphragms

It is easy to visualize the diaphragm on the right in expiration and inspiration, to measure thickness and also to assess diaphragmatic excursion during inspiration. The liver forms an acoustic window. On the left, the diaphragm can be seen at the level of the lower pole of the spleen. Depending on the filling status of the gastric fundus, the medial side can also be brought into view. Deep inspiration is problematic. Here, only the lateral parts close to the transducer are visible. It is difficult to assess the diaphragm excursion on the left side. Assessment of diaphragm excursion during deep breathing (in a sitting position) was only possible in 42% of cases [14]. While the thickness of the right half of the diaphragm could be measured in 95% of the attempts in mechanically ventilated patients, this was not consistently possible on the left side [7]. 

Boussuges et al. did not find any differences between the thickness of the right and left hemidiaphragms. The right to left ratio in end-expiration was ∼1. There was no significant difference for both genders: (1.1 ± 0.2 [range 0.7–1.5] in male and 1.1 ± 0.3 [range 0.6–1.6] mm in female [8]. In the study by Yamada et al., the diaphragm was significantly thicker on the right than on the left. The thickening fraction did not differ in both diaphragm hemispheres [14]. This is important for the differential diagnosis of dysfunctions. The upper limit of normality in the difference of the end-expiratory thickness of the two hemidiaphragms was 0.9 mm in men and 1 mm in women [8]. There may be small differences in TF_di_ between both diaphragm hemispheres in an individual healthy person. This difference was 13 ± 11% [LLN-ULN 0–34] in men and 16 ± 13% [LLN-ULN 0–41] in women. However, in healthy subjects, the TFdi could be identical for both diaphragm halves; in individual cases, the difference was up to 54% [8].

##### 3.3.6. Reproducibility of Ultrasound Measurements

The diaphragm is a narrow muscular structure only a few millimeters thick. Small measurement errors in the tenth of a millimeter range can lead to double-digit percentage deviations. For this reason, the diaphragm should be visualized with a high-resolution linear transducer when measured. Since the diaphragm is of different thickness in different areas, the measurement location should be marked for repeated measurements. A study on reproducibility showed that this was better with experienced examiners who were trained in diaphragm ultrasound. For less trained examiners, M-mode proved to be more accurate [10]. In healthy subjects, the TF_di_ with a cutoff of 36% proved to be a reliable parameter to rule out dysfunction even with less experienced examiners [10]. However, using M-mode for diaphragmatic thickness measurement could potentially introduce errors. Different subcostal transducer positions can lead to different diaphragm thicknesses. Untrained examiners, in particular, can achieve highly variable results. However, the advantage of M-mode is visualization of the diaphragm amplitude. For thickness measurement, the intercostal approach between the 8th and 10th ribs is preferred, but this requires appropriate instruction and supervision. After appropriate training, a high level of intraobserver and interobserver agreement can be achieved. In the study by Dhungana et al., two examiners were trained by a radiologist in the measurement of intercostal diaphragm thickness in three training sessions of 30 min each [20]. High agreement was achieved with an intraclass correlation coefficient for intraobserver variability of 0.986 (95%CI: 0.979–0.991) (*p* value of <0.001) and for interobserver variability 0.987 (95%CI: 0.949–0.997) (*p* value of <0.001) [20]. The authors concluded that the measurement of diaphragm thickness can be carried out reliably by intensive care physicians, after brief training. Other investigators also achieved an intraobserver variability < 10% after three to five training sessions of 15 min each [21]. 

##### 3.3.7. Advantages and Limitations of Diaphragm Ultrasound

The main advantage of ultrasound is that it is widely available, is cost-effective and can be performed anytime, anywhere, including in acute situations and on mechanically ventilated patients in the intensive care unit. No high-end ultrasound equipment is required for diaphragm ultrasound.

The various parameters mentioned above can be measured and derived using ultrasound, and diaphragmatic movement can be assessed. However, it is not possible to view the entire diaphragm. The posterior and medial parts, in particular, are more difficult to see. For this reason, diaphragmatic hernias or injuries cannot be ruled out using US. Obesity and anasarca can impair visualization of the diaphragm. Thickness measurements must be taken at the same position across examinations, as the diaphragm has different thicknesses in different areas. As the diaphragm is only a few millimeters thick, deviations of tenths of a millimeter can lead to a two-digit percentage deviation. A lack of experience leading to incorrect measurements at the wrong positions can result in deviating values. In mechanically ventilated patients, inspiration and expiration are determined by the machine. The examiner must find the right moment for measurements. Untrained, inexperienced examiners could make incorrect measurements resulting in significant error.

##### 3.3.8. Further Imaging

Other imaging procedures for assessing the diaphragm include chest X-ray, X-ray fluoroscopy, CT and (dynamic) MRI. While ultrasound, X-ray fluoroscopy and dynamic MRI are dynamic procedures, chest X-ray and CT are static procedures that do not allow function to be assessed. Chest X-ray is often an initial imaging modality that reveals unilateral or bilateral diaphragmatic eventration. Diaphragmatic movement can be visualized with X-ray fluoroscopy. This allows conclusions to be drawn regarding paralysis or paradoxical movement. CT allows an assessment of the entire diaphragm and the diagnosis of hernias and diaphragmatic injuries. CT is generally readily available but has the disadvantage of radiation exposure. CT is not suitable for assessing diaphragmatic function in critically ill and mechanically ventilated patients or in respiratory insufficiency during COPD exacerbation. CT is the modality imaging of choice in trauma patients to rule out diaphragmatic injury and incarcerated abdominal organs. The advantage of MRI is excellent evaluation of soft tissue structures. Dynamic MRI can also assess diaphragmatic excursion. However, MRI is not widely available, is more costly and is reserved for specialized questions. In the differential diagnosis of dyspnea, radiological procedures are also used to assess lung parenchyma. 

## 4. Deviations from a Healthy Diaphragm

### 4.1. Diaphragm Dysfunction

The diaphragm is the most important respiratory muscle; dysfunction leads to impaired contractility and ventilation. For patients, this means that diaphragmatic dysfunction causes orthopnea or dyspnea under physical strain, sleep disturbances and hypersomnia. Nocturnal hypoventilation and gastroesophageal reflux are also possible. Obesity and concomitant cardiac and pulmonary disease can worsen the severity of symptoms [4,22].

Any muscular disease or neurogenic disorder that affects the innervation of the diaphragm can cause diaphragm dysfunction [4,22]. Cardiopulmonary surgery, abdominal surgery, severe internal diseases, mechanical ventilation and trauma are among other causes of diaphragmatic dysfunction. The severity of the dysfunction can vary greatly. It includes diaphragmatic eventration, weakness or complete paralysis. Reduced, absent or paradoxical diaphragm movements indicate diaphragm dysfunction. 

**Diaphragmatic eventration** is a significant and abnormal elevation of the diaphragm. The attachment of the diaphragm to the sternum, ribs and dorsolumbar spine is preserved. In contrast to diaphragmatic hernias, the muscle continuity also remains intact.

***Unilateral involvement:*** Unilateral elevation can be an incidental finding in radiological diagnostics (chest X-ray, thoracic CT). Patients may be asymptomatic or exhibit the aforementioned symptoms. Patients with unilateral diaphragmatic elevation or dysfunction often have respiratory sleep disorders (fatigue, daytime sleepiness, snoring and apnea) [22]. For this reason, some authors recommend diagnostics in the sleep laboratory to identify patients with diaphragmatic elevation or paralysis [23,24].

Injury to the phrenic nerve is the main cause of unilateral diaphragmatic paralysis. This can be caused by thoracic and cervical surgery, ablation in atrial fibrillation or inflammatory mediastinal and intrathoracic processes as well as nerve compression due to space-occupying masses [17,25]. In the case of unilateral diaphragmatic elevation, pathological abdominal processes such as hepatomegaly and tumors should be excluded by ultrasound.

In patients with unilateral diaphragmatic paralysis, absent or paradoxical excursion (i.e., a cranial shift) is detected when examining diaphragmatic movement using M-mode US during resting breathing. Voluntary sniffing is used to detect paradoxical movements that indicate hemidiaphragmatic paralysis. During quiet breathing, a paradoxical diaphragmatic movement can be seen at the beginning of inspiration. During sniffing, there is a passive cranial displacement of the paralyzed hemidiaphragm. This paradoxical movement can be detected on ultrasound, especially M-mode. In the M-mode curve, an opposite movement can be seen compared to the nonparalyzed side [17,26,27]. On the healthy side, large diaphragmatic excursions can be seen as a compensatory mechanism for unilateral diaphragm paralysis [26]. The thickness of the paralyzed diaphragm hardly increases or even becomes thinner during inspiration [9].

***Bilateral involvement***: Patients with bilateral involvement are usually symptomatic and have dyspnea or orthopnea. If the diaphragm fails, the remaining respiratory muscles have to do all the work to compensate. The entire inspiratory work of breathing is performed by the contraction of the external intercostal muscles and the accessory muscles (M. sternocleidomastoideus, M. scalenus), which expand the chest and create negative intrathoracic pressure. This pulls the diaphragm as well as the abdominal viscera towards the thorax, which in turn leads to abdominal negative pressure. This causes paradoxical diaphragm and respiratory movements. Instead of expanding during inspiration, the thorax contracts.

If dyspnea in patients cannot be explained by cardiopulmonary causes, diaphragmatic dysfunction should also be considered in the differential diagnosis. The diagnosis of diaphragmatic dysfunction is based on static and dynamic imaging examinations as well as lung function tests and tests to stimulate the phrenic nerve [28]. Static methods assess the position, thickness and shape. These include the chest X-ray [29], B-mode ultrasound and CT [30]. The dynamic methods assess the movements and contractility of the diaphragm. In addition to M-mode US [26,27], this can be performed using fluoroscopy [29,31] or dynamic MRI [32].

***Diagnostic criteria for diaphragmatic paralysis*** include end-inspiratory thickness < 2 mm and TF_di_ < 20% during deep breathing (DB) [1,4,9]. A diaphragmatic amplitude < 2 cm in quiet breathing (QB) is considered a criterion for diaphragmatic dysfunction [4]. In the case of paralysis, the diaphragm thickness does not increase or increases only slightly during inspiration. A TF_di_ < 20% is indicative of diaphragmatic paralysis [9]. However, there are also healthy individuals in whom the diaphragm thickness increases by less than 10% or does not change at all during QB [8,14]. In one study, up to a third of the healthy test subjects had no to minimal diaphragmatic thickening during quiet breathing [33]. For diaphragmatic paralysis evaluation, assessing diaphragm thickness during deep breathing is more optimal. Using the percentage of thickening during deep inspiration is considered a better tool for detecting hemidiaphragmatic paralysis. The TR_di_ is recommended for detecting diaphragmatic paralysis. It is 2.1–2.2 [3,8]. For the thickening ratio, the lower limit of normality was 1.4 in men on both sides. In women, the lower limit of normality was 1.4 on the right side and 1.5 on the left side [8]. Ultrasound can be used to monitor the progress of diaphragm paralysis. Diaphragmatic paralysis is reversible in some patients. In 11/ 16 patients with different genesis of unilateral or bilateral diaphragm paralysis, this process functionally regressed in an observation period of 60 months using B-mode US. The mean time course was 14.9+/− 6.1 months [34].

### 4.2. Critically Ill Patients

#### 4.2.1. Noninvasively Ventilated Patients

Critically ill patients with respiratory insufficiency may have diaphragmatic dysfunction. In the study by Barbariol et al. [35], this was the case in 42% of patients. The proportion of diaphragmatic dysfunction was 1.9 times higher in postsurgical patients compared to nonsurgical patients. 

Diagnosis of diaphragm dysfunction by ultrasound may guide respiratory management decisions such as ventilation and need for ICU-level care.

The response to noninvasive ventilation (NIV) therapy was lower in patients with diaphragmatic dysfunction (60%) than in patients without diaphragmatic dysfunction (70%). With a cutoff of 1.37 cm of ultrasound diaphragm excursion as a potential predictor of NIV response, sensitivity and specificity were a modest 58% and 62.5%, respectively [35]. The high proportion of diaphragm dysfunction in intensive care patients with respiratory insufficiency is noteworthy. Diaphragm dysfunction after cardiopulmonary surgery, organ transplants, abdominal surgery, as well as serious internal diseases can be considered a form of organ failure [35]. Bedside ultrasound for diaphragm evaluation and clarification of the multifactorial etiology of respiratory insufficiency is easily possible. Consideration could be given to performing diaphragm ultrasound as an initial examination as part of the status assessment for patients with cardiopulmonary insufficiency or neurovascular or neuromuscular disorders on admission to the intensive care unit or intermediate care. If diaphragmatic dysfunction is present, NIV may be less successful.

#### 4.2.2. Invasively Mechanically Ventilated Patients

Ultrasonographic examination of the diaphragm allows for noninvasive measurement of diaphragmatic thickness and the degree of diaphragmatic thinning in patients receiving mechanical ventilation (MV). Diaphragm thickening reflects muscular contraction and not passive inflation in mechanically ventilated patients [7]. In patients who are mechanically ventilated, an early decrease in diaphragmatic thickness is rapidly seen [36,37,38]. Grosu et al. discovered a 6% per day decrease in diaphragmatic thickness. This process begins within 48 h after the start of mechanical ventilation [36]. Schepens et al. reported a decrease in diaphragm thickness of up to 32%. The greatest thickness loss occurred during the first 72 h of mechanical ventilation. The degree of decrease in diaphragm thickness was associated with the duration of mechanical ventilation [37]. Patients with prolonged weaning often have diaphragmatic dysfunction. These patients are ventilated longer and treated in intensive care units [39]. Measurement of diaphragmatic thickness at the beginning of mechanical breathing and then after 48 and 72 h may provide useful information. Atrophy could suggest a likelihood of delayed weaning.

Marques et al. investigated, in a meta-analysis, to what extent diaphragm ultrasound predicts the success of mechanical ventilation weaning in critical care [40]. Some further studies have found a link between reduced diaphragmatic excursion and diaphragmatic thickening fraction due to mechanical ventilation and weaning difficulties [41,42]. The results are not entirely conclusive. In summary, both ultrasound indices, diaphragm excursion (DE) and diaphragm thickening fraction, showed good predictive power for successful MV clearance in different populations in this meta-analysis. However, TF_di_ appeared to be the most accurate method for assessing diaphragm function in patients with MV. DE seems to be reserved for patients in whom no respiratory support is available. The optimal cutoff values were reported to be 1.0 to 1.4 cm for DE and 30–36% for TF_di_ [40].

#### 4.2.3. Neurovascular Diseases/Stroke

Reduced diaphragmatic amplitude and reduced diaphragmatic thickness during both inspiration and expiration on the affected hemiplegic side have been reported in some poststroke patients with hemiplegia. In the study by Cohen et al., this applied to 4/8 (50%) of patients [43]. In right-sided hemiplegia, the respiratory muscles were more severely impaired than in left-sided hemiplegia [44]. In the study by Liu et al., the incidence of diaphragmatic dysfunction during deep breathing was 46.67% in hemiplegic patients after stroke in the convalescent period. The paralyzed diaphragm was severely impaired, and the mobility of the paralyzed diaphragm was significantly reduced during deep breathing. In right-sided hemiplegia, the nonparalyzed side also showed significantly reduced diaphragmatic mobility [45]. A sonographic evaluation of diaphragmatic function appears useful for initiating appropriate longer-term physiotherapy measures to prevent pulmonary complications.

#### 4.2.4. Neuromuscular Diseases

Diaphragmatic ultrasound can be used to assess the contribution of diaphragmatic dysfunction or paralysis to respiratory failure in patients with various neuromuscular diseases. These include amyotrophic lateral sclerosis, multiple sclerosis, poliomyelitis, Duchenne muscular dystrophy, myasthenia gravis, Guillain–Barre syndrome and others [46]. 

In ***amyotrophic lateral sclerosis (ALS)***, the disease progresses to a weakness of the diaphragm, which is a major cause of respiratory failure. In patients with ALS, respiratory function testing may be difficult if there is also weakness of the fascial muscles. In this case, relevant diaphragm dysfunction can be used as an indication for mechanical ventilation support [47]. An important parameter is ΔTmax. This is the ratio of the thickening fraction between tidal volume and maximal lung capacity. A value of ΔTmax > 0.75 increased the risk of needing noninvasive ventilation (NIV) (HR = 5.6, *p* = 0.001) and of dying (HR = 3.7, *p* = 0.0001) compared to patients with lower values. The authors estimated that ΔTmax > 0.75 appears to correlate slightly better with initiating NIV within 6 months than with FVC < 50% predicted [47].

In the study by Spiliopoulos et al., only the percentage of thickening (TF_di_) correlated with the score on the revised ALS Functional Rating Scale. TF_di_ had the highest accuracy in predicting diaphragmatic dysfunction and the need to start noninvasive ventilation (NIV). A TF_di_ cutoff value of 0.50 was a sensitive threshold for NIV [48].

In children with ***Duchenne muscular dystrophy (DMD)***, it has been described that the diaphragm was thicker but did not show any significant increase in thickness at end-inspiration. This corresponds to the constellation of pseudohypertrophy [49] and emphasizes that the assessment of contraction is important for the assessment of function. In patients with Duchenne muscular dystrophy (DMD) with a wheelchair, diaphragmatic movements on deep inspiration and TF_di_ were reduced. Inspiratory diaphragmatic movements, expiratory diaphragm thickness and TF_di_ were inversely correlated with age [50].

### 4.3. Pulmonary Diseases

***Chronic obstructive pulmonary disease (COPD)****:* In patients with COPD and mild to moderate obstruction, the mean diaphragm thickness was reduced compared to controls. In severe COPD, the diaphragm thickness in expiration was increased compared to healthy subjects (3.7 mm versus 2.9 mm) [51]. The reduced diaphragmatic thickness is explained by thinning and shortening of the diaphragm due to hyperinflation [51]. There is no precise explanation for the thickening of the diaphragm in severe COPD. Pseudohypertrophy is conceivable. It has been described that patients may develop some adaptations in the diaphragm muscle, such as collagen accumulation. Diaphragmatic movement was reduced in patients with mild obstruction. In patients with moderate and severe obstruction, however, diaphragmatic movements were significantly increased compared to controls [51].

In a study of 54 COPD patients, diaphragmatic mobility was lower than in healthy subjects. Diaphragmatic mobility correlated positively with the distance covered in a six-minute walk test, while it correlated negatively with dyspnea on exertion [52]. 

In patients with exacerbated COPD, diaphragmatic excursion was a predictive parameter for the success or possible failure of NIV therapy. Patients with NIV success had greater diaphragm excursion before therapy (1.92 [1.22–2.54] cm versus 1.00 [0.60–1.41] cm) after one and two hours than those with failure. Diaphragm thickness and thickening fraction were similar and had no predictive value. However, the left-sided diaphragm thickness improved after 2 h in subjects with successful NIV, while no changes were observed over time in NIV failures. It is unclear why there were no other differences in diaphragm thickness and TFdi in this study population, as the investigators were sufficiently trained. In contrast to the study by Kocyigit et al., no subjects fell in the range with a TF < 20% [53]. In the study of Kocyigit et al., POCUS was performed in the emergency room on patients with exacerbated COPD. The thickening fraction was used to predict NIV failure. A diaphragm thickening fraction of less than 20% during spontaneous breathing was used as a cutoff value. NIV failure was found in 73% with diaphragm dysfunction and in only 4.4% without. In predicting NIV failure, diaphragm dysfunction had a sensitivity and specificity of 84.6% and 91.5% [54], respectively. Even though these results are contradictory, it is clear that diaphragmatic US offers interesting approaches and support for making treatment decisions even in patients with exacerbated COPD. For patients admitted to the emergency department with acute dyspnea, a diaphragmatic ultrasound excursion of less than 2.3 cm was associated with the need for NIV [55]. A significant reduction in diaphragmatic ultrasound excursion may signal a likelihood of failed weaning and a longer duration of invasive mechanical ventilation [56]. The measurement of diaphragm thickness and excursion is not part of the various POCUS protocols. However, consideration should be given to evaluating this in patients with COPD exacerbation in the emergency department. 

In addition to COPD therapy, the consequence of diaphragm dysfunction is additional targeted diaphragm therapy: the effectiveness of diaphragm-targeted rehabilitation therapies has been proven and should be included in the lung rehabilitation strategy for COPD [57]. Related rehabilitation therapies include diaphragmatic breathing, diaphragm-related manual therapy and electrical stimulation of the phrenic nerve. Electrical stimulation of the phrenic nerve is reserved for patients with severe disease. These therapies have a positive effect on diaphragm function, lung function, dyspnea and physical resilience [57].

***Bronchial asthma***: For bronchial asthma, a moderately restricted inspiratory force and a slight increase in diaphragm thickness have been described [58].

***Cystic fibrosis***: In a study by Pinet et al., patients with cystic fibrosis, severe respiratory failure and malnutrition had diaphragmatic weakness but no muscle atrophy. Diaphragm thickness was greater than that of healthy controls. This was explained by hypertrophy due to respiratory muscle training [59]. In the study by Dufresne et al., patients with cystic fibrosis had thicker diaphragms and greater muscle strength than control subjects [60].

***Interstitial lung disease***: Patients with interstitial lung disease (ILD) and pulmonary fibrosis typically suffer from dyspnea and exercise intolerance. Dysfunction of the respiratory muscles is one of the causes. Patients with interstitial lung disease had a mean diaphragmatic mobility that was comparable during quiet breathing, although it was significantly lower in the patients during deep breathing (4.5 ± 1.7 cm vs. 7.6 ± 1.4 cm; *p* < 0.01) compared to controls. The diaphragm was thicker at end-expiratory time but without significant contraction at end-inspiratory time. Thus, the thickening fraction was limited in patients with interstitial lung disease [61]. These results were confirmed in patients with fibrotic interstitial lung function. They correlated with the degree of dyspnea, limitations in exercise tolerance, quality of life and lung function [62]. 

In a study collection of interstitial lung diseases, 29% had diaphragmatic dysfunction. These were patients with idiopathic pulmonary fibrosis (IPF) and connective tissue disease (CTD-ILD). In CTD-ILD, diaphragm displacement and end-inspiratory thickness were lower as compared to IPF. In contrast, in CTL-ILD diaphragmatic dysfunction was more prevalent compared to IPF and controls (37% vs. 22% vs. 7%) [63]. 

In patients with mild to moderate restrictive interstitial pulmonary fibrosis, diaphragmatic function did not appear to be impaired. Other authors found no association between diaphragmatic function, respiratory function parameters and the extent of fibrosis in mild to moderate interstitial fibrosis [64].

### 4.4. COVID-19

Various mechanisms could lead to diaphragmatic weakness in COVID-19 patients. These were critical illness and mechanical ventilation but also neuromuscular disorders and severe lung disease with the development of fibrosis and (fibrotic) intestinal lung disease. Diaphragmatic dysfunction in all these diseases could worsen in COVID-19 dysfunction with critical illness and mechanical ventilation [65,66]. The extent to which COVID-19 causes damage to the phrenic nerve has not been proven with certainty. Patel et al. estimate that diaphragmatic ultrasound was helpful in patients with COVID-19 who had difficulty weaning from ventilation and in other patients who were weaned from prolonged ventilation and continued to suffer from dyspnea and oxygen deficiency [66]. Dyspnea in patients with COVID-19 requires complex diagnostics with assessment of lung impairment and possible fibrosis on CT and respiratory function tests. Diaphragmatic ultrasound makes it possible to evaluate diaphragmatic dysfunction. This can be repeated over time to assess the dynamics of deterioration or recovery. Diaphragmatic dysfunction with or without pulmonary involvement can be an indication for (nighttime) noninvasive ventilation [66].

### 4.5. Congenital and Acquired Diaphragmatic Hernias and Traumatic Injuries

Diaphragmatic hernias are congenital or acquired, the latter are usually posttraumatic after abdominal trauma. Rare causes include surgery or pregnancy. Congenital diaphragmatic hernias (CDHs) develop during embryogenesis when the diaphragm does not form properly. During fetal development, this leads to the ingestion of abdominal organs into the thorax with impaired lung development. The clinical severity of CDH depends on the extent of lung tissue displacement, lung hypoplasia and the severity of pulmonary hyperplasia and pulmonary hypertension. In the California population, CDH had a prevalence of 3.30 per 10,000 total births [67]. Among >24 million births in China, the prevalence of CDH was 1.8/10,000 [68]. In an Australian study, 35% of live-born infants with CDH died before referral or transport to an appropriate tertiary care center. Only 52% of live-born infants, 32% of all cases and 16% of all prenatally diagnosed cases survived [69]. Prenatal diagnosis and referral to an appropriate center are therefore extremely important. 

A distinction is made between posterolateral hernias (Bochdalek hernias) and anterior parasternal Morgagni hernias via the Morgani foramen. Posterolateral hernias (Bochdalek hernias) are the most common. They account for more than 80% and are usually left-sided (85%), less frequently right-sided (13%) or bilateral (2%) [67]. The Morgagni hernia is correspondingly rare. It occurs as a result of maldevelopment of the sternal attachments to the diaphragm [67]. CDH in adults is much rarer. Diagnosis is usually made in connection with symptoms caused by the hernia (86%), pain (69%) and pulmonal symptoms [70,71,72,73]. 

Both CDH and posttraumatic ruptures occur more frequently on the left side [70,74,75]. This may be due to the protective space-occupying effect of the liver. Incarceration of the liver into the thorax is an extremely rare event [76].

The diagnosis of hiatal hernias is usually a finding in adults during gastroscopy. Very large hernias can lead to a partial displacement of the stomach into the thorax, the (partial) thoracic stomach. This finding can also be seen sonographically if there is a suspicion. These patients often become conspicuous due to gastrointestinal bleeding or anemia. Paraesophageal hernias are noticeable during gastroscopy or CT.

**Figure 8:**
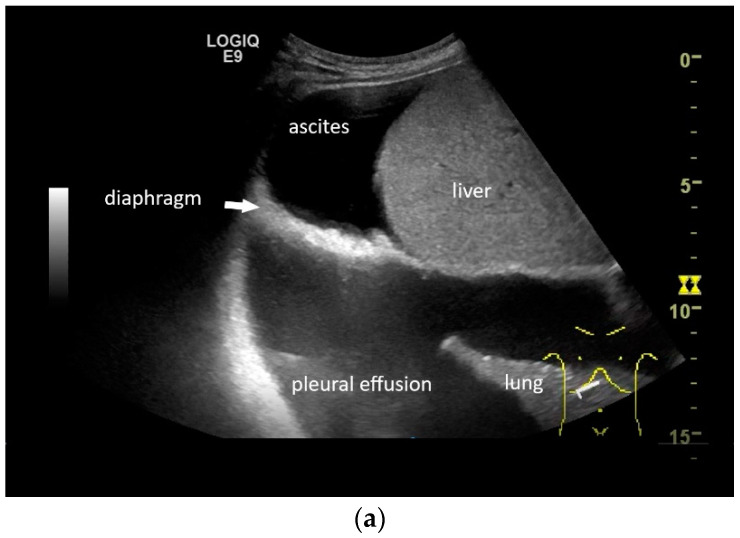

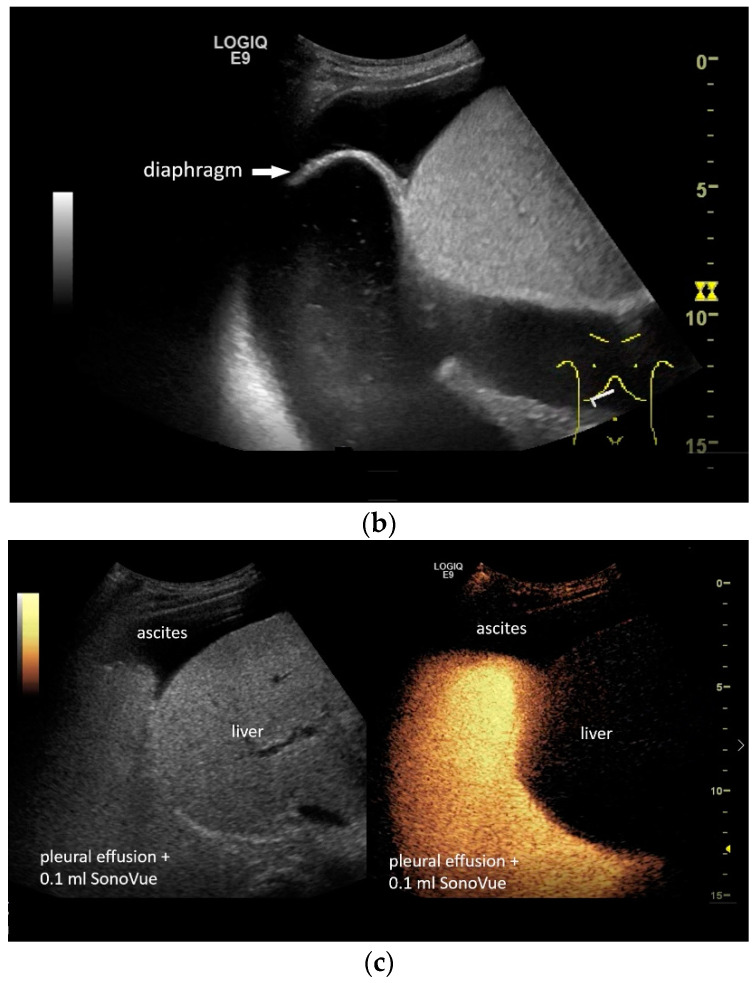
In pleural effusion and ascites, diaphragmatic movements can be seen. In expiration (**a**) and during deep inspiration, the diaphragm moves towards the abdominal cavity (**b**). Intracavitary contrast administration with SonoVue (off-label use) was performed for ascites and right pleural effusion under the suspicion of a fistula between the pleura and abdomen with recurrent right-sided pleural effusion. However, there was no transfer of the contrast medium from the pleural effusion into the ascites (**c**).

If a diaphragmatic fistula is suspected in cases of ascitic decompensated liver cirrhosis and concurrent unilateral pleural effusion, it is possible to use intracavitary CEUS to demonstrate the passage of the contrast agent from one cavity to the other. However, this is off-label use and usually the last diagnostic step (Figure 8).

Injury to the diaphragm in the event of trauma is very difficult to diagnose sonographically. Indicative findings can be a displacement of abdominal organs into the thorax but also a disturbed diaphragmatic movement following trauma. Sharifi et al. compared the results of focused assessment with sonography for trauma (FAST) with those of laparoscopy in patients with penetrating thoracoabdominal trauma. FAST had a sensitivity of 50% with a specificity of 100% [77]. However, the sonographically suspicious findings should not be ignored. High-energy trauma and penetrating injuries are the domain of CT and laparoscopy, but the FAST examination is used to set the course. If diaphragmatic injury is not recognized in acute trauma, incarceration and strangulation of the innervated organs can occur, significantly increasing mortality. CT has also been shown in various studies to have varying degrees of accuracy in the diagnosis of diaphragmatic injuries. The sensitivity and specificity of CT in the diagnosis of diaphragmatic injuries are reported to be 84% and 95%, respectively [78]. However, there are other studies in which the accuracy was lower [79,80,81]. Diagnostic laparoscopy therefore remains the gold standard. MRI is mainly employed for evaluation of congenital and posttraumatic hernias. However, there are also some studies in which diaphragmatic trauma was investigated using MRI [82,83]. MRI is superior to CT in the visualization of soft tissue structures. Dynamic MRI has the advantage of permitting assessment of diaphragmatic movements. Using the coronal and sagittal planes, it is possible to assess the diaphragm under normal as well as pathological conditions [83]. However, many hospitals are not equipped to perform acute trauma MRI. The usual disadvantages of CT are radiation exposure and the potential nephrotoxicity of contrast agents, but MRI is more costly, with its use possibly limited by resources. Additionally, pacemakers, defibrillators and some metal implants are contraindications.

The diagnosis of a diaphragm injury by ultrasound requires a high level of experience. A displacement of the stomach and colon into the left hemithorax is typical [74,84,85]. It may be indicative that the spleen is not visible because it is overlaid by colonic structures and their artifacts. Ultrasound is not able to rule out diaphragmatic injury. 

Imaging diagnosis of diaphragm hernias is made by chest X-ray if an abdominal organ such as the stomach or intestine is visible in the chest and by CT. The standard diagnostic test is CT. CT is used to diagnose the presence of the hernia, its location and size and to determine the prolapsed organs. If there is a history of trauma in the presence of symptoms and visible abdominal organs in the thoracic cavity, the diagnosis is quite clear. Patients may also be asymptomatic, and the diagnosis may be made much later [86]. An important differential diagnosis is unilateral diaphragmatic eventration/elevation, which occurs more frequently on the right side and is usually asymptomatic or correlated with dyspnea and in which the diaphragmatic muscle is intact. 

### 4.6. Tumors and Lesions

Tumors of the diaphragm are very rare. These are primary benign and malignant tumors and secondary tumors as metastases from tumors of the thoracoabdominal cavities. Apart from cysts, primary diaphragm tumors are predominantly mesenchymal tumors. Baldes and Schirren analyzed 200 case reports of diaphragm tumors [87]. By far the most common benign tumor was lipoma. Other benign tumors are neurofibroma, angiofibroma, schwannoma, fibroma and hemangioma, for which there are several reports. There are individual case reports for a large number of other benign mesenchymal tumors [87]. Manifestations of echinococcus cysts are also mentioned although very rarely [88,89]. Endometriosis manifestations are usually diagnosed surgically in the case of catamenial pneumothorax [90,91,92].

The most common malignant tumors are rhabdomyosarcoma and fibrosarcoma followed by leiomyosarcoma, undifferentiated sarcoma and Yolc sac tumor. There are individual case reports for other malignant sarcomas [87]. Secondary tumors are more common. Important primary sites are pulmonary carcinomas, infiltration of mesothelioma from the parietal pleura into the diaphragm, residuals of germ cell tumors or sarcomas and metastases of malignant thymoma [87]. In a retrospective study by Guo et al., all 18 primary diaphragmatic tumors were benign, again with a predominance of cysts and lipomas. The cysts were of both bronchogenic and mesenteric origin [93].

The sonographic diagnosis of diaphragmatic tumors depends primarily on the visibility of the diaphragm. Experience has shown that the right hemidiaphragm is easier to see than the left side. Nevertheless, the diaphragm cannot be completely imaged on the right side either. Pleural effusion, ascites and large masses can improve the assessment, while obesity and small tumors make sonographic assessment more difficult. The tumors described show their typical characteristics in ultrasound. It is probably easiest for cysts. Otherwise, the domain for diagnosing diaphragmatic tumors is CT. 

### 4.7. In Sports

In sports, diaphragmatic ultrasound can be used to assess diaphragm thickness and respiratory function to evaluate training results and adapt training. Both diaphragm thickness and mobility are influenced by the type of sport [94,95]. 

Diaphragm thickness is positively correlated with anaerobic power. Anaerobic power describes the maximum power that is exerted during a very short, very intensive effort of a few seconds. This means that greater diaphragm thickness can improve performance in certain high-intensity sports. The diaphragm thickness of elite powerlifters was greater than that of controls [96]. Inspiratory muscle training resulted in significant diaphragmatic hypertrophy and increased inspiratory muscle strength in paralympic athletes with cervical spinal cord injury [97]. Aerobic performance appears to be related to cardiovascular efficiency and oxygen transport rather than diaphragm thickness. Endurance sports do not appear to cause the same morphological changes in the diaphragm as anaerobic sports [95]. 

## 5. Controversies

Ultrasound is a widely used imaging technique for a variety of indications across almost all organ systems. However, in reality, diaphragmatic ultrasound is not yet widely available. Who should perform the examination? Pulmonologists, cardiologists, anesthetists, radiologists or the specialist with the highest ultrasound level? Or is it sufficient to have knowledge at the POCUS level? The authors believe it should be a physician who performs a quality ultrasound examination and understands the clinical context. Measurements of the diaphragm require training and supervision to achieve acceptable accuracy and reproducibility.

Which measurement position is preferable? In the EXpert consensus on diaphragm ultrasound in the critically ill (EXODUS) guidelines by Haaksma et al., M-mode is described as the best method to learn [4]. However, clinical experience suggests that different M-mode curves are derived in different subcostal positions. The advantages of M-mode measurement are assignment of the curve to the respiratory cycle and measurement of the amplitude. An abdominal curved array transducer is required for M-mode measurement in the medioclavicular line. However, the diaphragm cannot always be well delineated under these conditions. B-mode measurement using an intercostal window has a higher spatial resolution. Therefore, the authors recommend B-mode measurement in the intercostal position with appropriate marking for follow-up. 

Should diaphragm ultrasound be mandatory for critically ill patients before NIV or mechanical ventilation? How would this influence the procedure? Would patients with diaphragmatic dysfunction be ventilated mechanically rather than by NIV? What is the outcome of patients with initial diaphragm dysfunction and mechanical ventilation? Diaphragmatic atrophy due to mechanical ventilation may predict delayed weaning. But how does this affect the treatment strategy? How does diaphragm ultrasound influence mechanical ventilation? Would other ventilation pressures be selected? In which situation do the parameters of diaphragm ultrasound influence the further procedure and outcome of the patient, and when is the decision based solely on the other clinical parameters? The authors suggest that these are open questions that require further study. According to Haaksma et al., ultrasound should be investigated as a screening method for detecting patient–ventilator asynchrony [4]. In addition, the role of diaphragmatic ultrasound in the effective titration of ventilator settings and the importance of diaphragmatic ultrasound in noninvasive ventilation (e.g., as a predictor of release from mechanical ventilation or for titration of support settings) should be the subject of further studies [4]. 

A reduced diameter below the LLN is a criterion for atrophy. However, thickening can be the result of hypertrophy/pseudohypertrophy or inflammatory edema. Further differentiation would be advantageous here. This could be an application for shear wave elastography or speckle tracking imaging.

## 6. Conclusions

The diaphragm is the main respiratory muscle. Diaphragm function can be impaired in various diseases, in critically ill patients, in mechanical ventilation, in neuromuscular and neurovascular diseases and also in some lung diseases. Diaphragmatic dysfunction can be the cause of unexplained dyspnea and impaired exercise tolerance. Diaphragmatic ultrasound can be used to objectively determine the extent to which dysfunction or paralysis of the diaphragm contributes to respiratory failure. It is possible to diagnose diaphragmatic dysfunction with B-mode and M-mode ultrasound. The most important parameters are the end-expiratory and end-inspiratory diaphragm thickness, the resulting thickening rate and thickening fraction and the amplitude of diaphragmatic movements. 

A diaphragm thickness of </=1.7 mm in females and 1.4 mm in males in a supine position or </=1.1 mm in females and 1.3 mm in males in a seated position are signs of atrophy. A diaphragm thickening fraction of <20% during DB is a criterion of dysfunction. A thickened diaphragm is the result of hypertrophy or pseudohypertrophy, for example, after exercise or in COPD, but can also be the result of inflammatory edema. The determination of these parameters requires training, and at least 40 supervised examinations should be completed.

The response to noninvasive ventilation is lower in patients with diaphragm dysfunction. Mechanical ventilation causes diaphragm atrophy, and thus, delayed weaning can be predicted. The diaphragm can be affected in patients with neurovascular diseases and especially hemiplegia. Special physiotherapeutic measures may be indicated to prevent respiratory complications. In neuromuscular diseases, relevant diaphragm dysfunction can be used as an indication for noninvasive ventilation or mechanical ventilation support. In patients with exacerbated COPD, diaphragmatic excursion is a marker of response to noninvasive ventilation. Ultrasound-assisted diagnosis of diaphragmatic dysfunction supports the indication for therapeutic procedures. 

Patients with COVID-19 infection may also have diaphragmatic dysfunction independent of diffuse lung disease. Follow-up checks can be carried out with ultrasound.

Despite the many potential advantages and uses of ultrasound, the entire diaphragm cannot be visualized. This is particularly important in cases of trauma with injury to the diaphragm or hernias. Therefore, this is the domain of CT. However, it is prudent to perform a quality diaphragm ultrasound examination in any patient with unclear dyspnea and a medical history that makes diaphragm dysfunction likely in order to carry out appropriate therapeutic measures.

## Figures and Tables

**Table 3:** Lower limit of normality (LLN).

Parameter	What Is Pathological?
LLN of diaphragm thickness at end-expiration—supine position	</= 1.7 mm in females and 1.4 mm in males [3,12]
LLN of diaphragm thickness at end-expiration—seated position	</= 1.1 mm in females and 1.3 mm in males [8]
ULN of difference of diaphragm thickness between the left and right sides	>/= 1 mm in females and 0.9 mm in males [8]
TR_di_ (normal value)	2.1–2.2 [3,8]
LLN of TR_di_	<1.4 in male on both sides <1.4 on the right side and 1.5 on the left side in females [8]
TF_di_	<20% during DB [9,17]
Diaphragm excursion	<20 mm during QB [4]

Legend: DB, deep breathing; QB, quiet breathing; TR_di_, thickening ratio; TF_di_, thickening fraction; LLN, lower limit of normality; ULN, upper limit of normality.

## Abbreviations

D, diaphragm; DB, deep breathing; DE, diaphragm excursion; FRC, functional residual capacity; LLN, lower limit of normality; QB, quiet breathing; TLC, total lung capacity; T_di_, diaphragm thickness; T_endexpi_, thickness end-expiratory; T_endinspir_, thickness end-inspiratory; TF_di_, diaphragm thickening fraction; TR_di_, diaphragm thickening ratio; ULN, upper limit of normality; ThFRC, thickness at functional residual capacity; ThTLC, thickness at total lung capacity.
